# Meiotic recombination in the offspring of *Microbotryum* hybrids and its impact on pathogenicity

**DOI:** 10.1186/s12862-020-01689-2

**Published:** 2020-09-17

**Authors:** Britta Bueker, Marco Alexandre Guerreiro, Michael E. Hood, Andreas Brachmann, Sven Rahmann, Dominik Begerow

**Affiliations:** 1 AG Geobotanik, Ruhr-Universität Bochum, Universitätsstraße 150, 44780 Bochum, Germany; 2 Department of Biology, Amherst College, 220 South Pleasant Street, Amherst, MA 01002 USA; 3 Biocenter of the LMU Munich, Genetics Section, Grosshaderner Str. 2-4, 82152 Planegg- Martinsried, Germany; 4 Genominformatik, Institut für Humangenetik, Medizinische Fakultät, Universität Duisburg-Essen, Hufelandstraße 55, 45122 Essen, Germany

**Keywords:** Experimental selection, Host-pathogen interaction, Hybrid speciation, Backcrossing, Host specialization, Effectors

## Abstract

**Background:**

Hybridization is a central mechanism in evolution, producing new species or introducing important genetic variation into existing species. In plant-pathogenic fungi, adaptation and specialization to exploit a host species are key determinants of evolutionary success. Here, we performed experimental crosses between the two pathogenic *Microbotryum* species, *M. lychnidis-dioicae* and *M. silenes-acaulis* that are specialized to different hosts*.* The resulting offspring were analyzed on phenotypic and genomic levels to describe genomic characteristics of hybrid offspring and genetic factors likely involved in host-specialization.

**Results:**

Genomic analyses of interspecific fungal hybrids revealed that individuals were most viable if the majority of loci were inherited from one species. Interestingly, species-specific loci were strictly controlled by the species’ origin of the mating type locus. Moreover we detected signs of crossing over and chromosome duplications in the genomes of the analyzed hybrids. In *Microbotryum*, mitochondrial DNA was found to be uniparentally inherited from the a_2_ mating type. Genome comparison revealed that most gene families are shared and the majority of genes are conserved between the two species, indicating very similar biological features, including infection and pathogenicity processes. Moreover, we detected 211 candidate genes that were retained under host-driven selection of backcrossed lines. These genes and might therefore either play a crucial role in host specialization or be linked to genes that are essential for specialization.

**Conclusion:**

The combination of genome analyses with experimental selection and hybridization is a promising way to investigate host-pathogen interactions. This study manifests genetic factors of host specialization that are required for successful biotrophic infection of the post-zygotic stage, but also demonstrates the strong influence of intra-genomic conflicts or instabilities on the viability of hybrids in the haploid host-independent stage.

## Background

Hybridization is now realized as a common and important evolutionary mechanism in the diversification of species, particularly for plant-pathogenic fungi. By the successful crossing of hetero-specific individuals, genetic material of both species is transmitted and the infusion of new allelic combinations and recovery from fixed genetic load loci can have marked impacts on fitness and adaptive potential. Molecular analyses have revealed that hybridization occurs in different multiple groups of fungal plant pathogens, e.g., *Microbotryum violaceum, Zymoseptoria pseudotritici, Cryptococcus neoformans* [1–4]. In particular, naturally occurring hybridization can lead to new pathogenic species, accelerated by new gene combinations and subsequent adaptive specialization.

Central to the ecology of pathogenic fungi is their adaptation to exploit the host environment, and there is broad variation in host specialization and adaptation among pathogens [5]. This form of adaptation to the host can strongly impact patterns of speciation [6, 7]. In contrast to generalists, which are able to infect several different often only distantly related host species, specialized, or host-specific, pathogens are highly adapted to one or few, often closely related, host species. In plant-pathogenic fungi, host specificity is often characteristic of the biotrophic lifestyle, where the fungi colonize and exploit living host tissues [8]. These biotrophic fungi often produce ‘effector’ proteins. Effector proteins are secreted by the fungi into the apoplast or even into the host cell, where they are reported to modify the host defenses, cell structure, metabolism and function [9]. Molecular analyses of diverse biotrophic species show that, besides the existence of effector proteins that are conserved among taxa, there can also occur species-specific effectors that are highly variable and can interact with host receptor molecules with arms-race dynamics [5, 10]. While pathogenicity determinants, including effectors, have been described for a reasonable number of fungal pathogens, the genetics and genomics for genes shaping host specificity seem to be more difficult to resolve [5]. Genome and secretome (i.e. all secreted proteins) analyses of fungal species with different lifestyles revealed a great repertoire of effector proteins that are common in fungi to promote virulence and interact with the host. However, the function of such effector proteins or their role in specialization is often unclear [5, 11].

The process of hybridization can combine adaptive traits of different pathogenic species [12]. There is growing recognition of the threat of hybrid pathogens, which arise in part due to the new combinations of specific effector repertoires found in the parental pathogen species [13]. Thus, hybrid pathogens have been observed to emerge, causing new diseases with altered host affinities [14, 15]. Moreover, there is molecular evidence from the study of natural and experimentally created hybrids for a role of particular genes involved in pathogenicity [13]. For example, the rust *Melampsora* × *columbia* is a hybrid species derived from *M. medusae* and *M. occidentalis* and evolved when a poplar host resistant to the two parental species was widely grown in California [16]. Under experimental evolution, the *Neurospora crassa* × *N. intermedia* hybrids have been used to gain a better understanding of genetics underlying traits of reproductive incompatibility between species [17]. Due to the importance to natural and agricultural ecosystems, in combination with tractability as genetic and genomic models, hybrid fungal pathogens offer strong potential to advance our understanding of the molecular basis of host range through experimental crosses and host-specific selection. Thus, especially in the light of host-driven selection, the host-pathogen interactions of hybrid fungi and the dynamics of their genome composition can reveal insight on host specialization in fungi.

The basidiomycetous genus *Microbotryum* includes a species complex of biotrophic plant pathogens causing anther-smut disease. Some species in this complex colonize and sporulate in the flowers of plants in the Caryophyllaceae family. Largely restricted to natural ecosystems, many *Microbotryum* species, including the group of anther smuts on members of the genus *Silene*, are specialized on one host species [18]. The pathogenic life-cycle and mating system proceed as follows. Infected host plants are sterilized through the replacement of pollen by diploid fungal spores and the inhibition of ovary development. After transmission from diseased to healthy plants, spores germinate and undergo meiosis, resulting in yeast-like haploid cells with two mating types a_1_ and a_2_. Mating types are determined by largely non-recombining mating type chromosomes that carry genes that are needed for conjugation, e.g., pheromone/pheromone receptor and mating type homeodomain genes [19]. Conjugation between cells with opposite mating types occurs and infectious structures are formed: dikaryotic hyphae with appressoria grow and enter the host, maintaining infection in meristems until, in floral development, the fungus sporulates in the anthers [20]. Although the genome and transcriptome of *Microbotryum lychnidis-dioiceae* have been recently analyzed, providing first insights into genetic features of its pathogenicity and life-cycle [21–23], the genetics of host specificity in *Microbotryum* are as yet poorly understood in relation to the group of related hosts and multiple pathogenic species.

Here, we use an experimental crossing approach to study traits of hybridization in relation to host adaptation in *Microbotryum*. Therefore, we combine infection studies using hybrids and backcrosses with the analyses of genomes of selected offspring. First, we use experimental hybrids and backcrosses to study hybridization events and the effects on infection ability, with special regards to the role of compatibility between mating type chromosomes from different species. Second, we compare the genomes of two highly host-specific *Microbotryum* species and describe characteristics of specialization on genomic and phenotypic levels. Third, we use the experimental hybrids and backcrosses to select for host-specificity loci, leading to the identification of candidate genes involved in host-specific virulence in *Microbotryum*.

## Results

### Infection rates and viability of the parental species, F1- and F2-hybrids

**Fig. 1:**
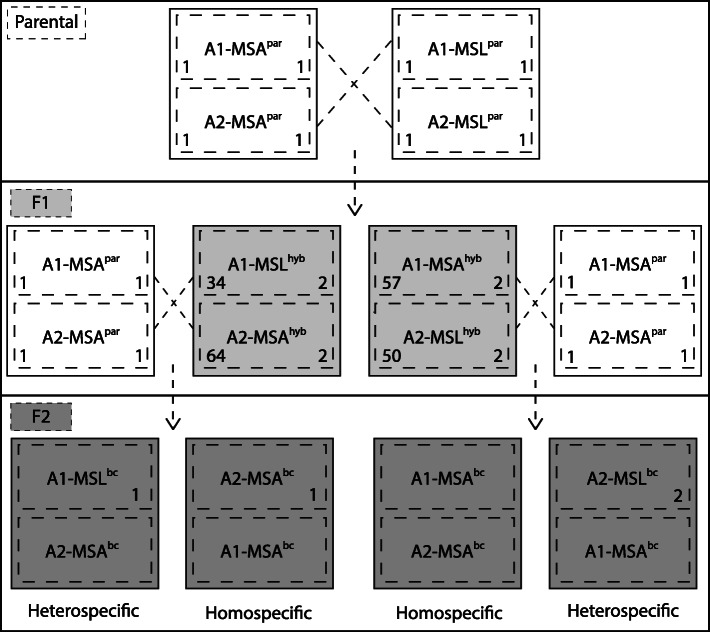
Crossing scheme that is used for the selection of host specificity loci in *Microbotryum*

**Fig. 2:**
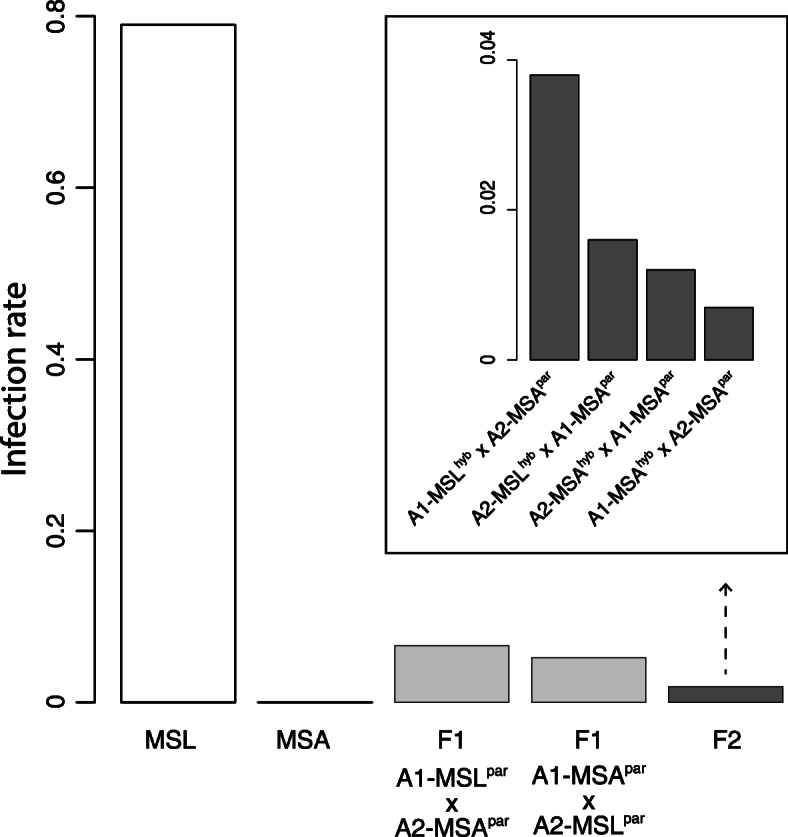
Infection rates of intra- and inter-specific crosses on the host *S. latifolia*

In order to estimate species-specific pathogenicity, infection rates for both parental *Microbotryum* species and hybrid offspring were estimated on the host *Silene latifolia* (Fig. 1). The *Microbotryum* species demonstrated strong host specificity upon experimental inoculation. From 50 *S. latifolia* plants that were inoculated with its endemic pathogen *Microbotryum lychnidis-dioiceae* (MSL), a proportion of 0.79 became diseased, while none of the 50 plants inoculated with *M. silenes-acaulis* (MSA) exhibited disease symptoms (Fig. 2). *Microbotryum* F1-hybrids caused disease but at rates lower than the *M. lychnidis-dioiceae* parental species. *Silene latifolia* plants inoculated with F1-hybrids between the *Microbotryum* species MSL and MSA, showed an infection rate of 0.06 on average, with plants inoculated using F1-hybrids where the a_1_ mating type chromosome derived from MSL (A1-MSL^par^ × A2-MSA^par^) showed only non-significant marginally higher infection rates (0.06) than F1-hybrids where the a_2_ mating type chromosome derived from MSL (A1-MSA^par^ × A2-MSL^par^) (0.05).

*Microbotryum* F1-hybrids were able to produce viable meiotic products that could successfully mate. While spores of each F1 type showed successful germination after one to 2 days on culture media, for the hybrid type A1-MSL^par^ × A2-MSA^par^ we were able to isolate 31 cultures for the a_1_ mating type (A1-MSL^hyb^) and 64 cultures for the a_2_ mating type (A2-MSA^hyb^) with positive conjugation ability. For the second hybrid type (A1-MSA^par^ × A2-MSL^par^) 46 a_1_ (A1-MSA^hyb^) cultures and 50 a_2_ cultures (A2-MSL^hyb^) were isolated with confirmed conjugation ability.

From plants inoculated with haploid mixtures that produce the backcrossed F2 pathogen generation, 5 to 19 plants were infected per treatment (infection proportion between 0.01 and 0.04). Those treatments where both mating types were derived from MSA (homospecific backcross) showed lower but not significantly differing infection rates (A1-MSA^hyb^ × A2-MSA^par^: 0.01, A2-MSA^hyb^ × A1-MSA^par^: 0.01) than treatments where one mating type was derived from MSL (heterospecific backcross) (A1-MSL^hyb^ × A2-MSA^par^: 0.04; A2-MSL^hyb^ × A1-MSA^par^: 0.02) (Fig. 2). Among the F2-backcrosses pathogens, the two heterospecific backcrosses A1-MSL^hyb^ × A2-MSA^par^ and A2-MSL^hyb^ × A1-MSA^par^ and the homospecific backcross A2-MSA^hyb^ × A1-MSA^par^ had successful germination of teliospores, while for the genotype A1-MSA^hyb^ × A2-MSA^par^ no successful germination of teliospores was observed. For the heterospecific backcross A2-MSL^hyb^ × A1-MSA^par^ haploid isolates were viable and isolated and sequenced as described above (Fig. 1).

### Sequencing of genomes and genetic distance

For molecular analyses of the two host-specific species and their hybrids, genomes were sequenced. For eleven of twelve genomes, the number of obtained reads varied from 4.4 to 5.7 million after pre-processing, except for one of the F1-hybrid isolate A1-MSL^hyb^ where only 101,986 reads were obtained.

The mapping of parental reads against the reference genome from MSL resulted in 0.99 of MSL reads that could be mapped, while only 0.92 of the MSA could be mapped. Assembly statistics of the MSL a_1_ and a_2_ genomes with the reference genome as target and the *M. silenes-acaulis* are in shown in Table 1. From the 7364 reference genes, 7320 could be detected in the MSL a_1_ genome and 7205 in the MSL a_2_ genome (average: 7263), while 7134 and 7127 were detected in the MSA a_1_ and a_2_ genomes (average: 7134), respectively (Table 1). Alignments between genome assemblies of MSL^par^ and MSA^par^ resulted in 5751 and 5433.

**Table 1:** Assembly statistics of the four parental genomes *M. lychnidis-dioicae* (MSL) (a_1_ and a_2_) and *M. silenes-acaulis* (MSA) (a_1_ and a_2_)

	A1-MSL^par^	A2-MSL^par^	A1-MSA^par^	A2-MSA^par^
Coverage X	33 X	30 X	26 X	29 X
Assembly size (Mb)	21.8	25.6	22.8	21.8
Contigs	4398	3458	3244	3724
Contigs N50	13,234	18,905	13,947	15,025
GC Content	54	54	53	53
Count of Genes	7320	7205	7167	7200
Count of Autosomal Genes	6838	6799	6579	6654
Count of NRR/ PAR Genes	333/99	274/99	253/97	270/98

*NRR* non-recombining regions, *PAR* pseudo-autosomal regions

homologues for A1-MSL^par^ vs A1-MSA^par^ and A2-MSL^par^ vs A2-MSA^par^, respectively, with an average nucleotide identity of 0.94 (Table 2). Genetic distance of hybrid genomes to parental genomes was estimated by differences in karyotypic electropherograms. Comparison of hybrid and parental genomes revealed that genome size and structure of all hybrids were similar to parental sizes and no apparent allopolyploidy or genome duplication occurred. However, pairwise electropherogram comparisons were more similar for mating type chromosomes in hybrid and parental strains derived from the same species (Table 2). Genetic distance based on nucleotide identity showed the same trend. For the hybrid genotypes A1- or A2-MSL^hyb^_,_ genetic distance to the MSL^par^ was lower than to MSA^par^, while for the hybrid types A1- or A2-MSA^hyb^ genetic distance to MSA^par^ was lower than to MSL^par^ (Table 2).

**Table 2:** Genetic distance between F1-hybrid genomes and the parental MSL and MSA genomes, based on a) karyotype analysis and b) single-nucleotide polymorphisms (SNPs)

Genetic distance	Karyotype		SNP	
	MSL^a^	MSA^a^	MSL^a^	MSA^a^
MSL^par^	0	1	< 0.1	3.437
MSA^par^	1	0	3.433	< 0.1
A1-MSL^hyb^	0.863	0.976	< 0.1	3.431
A2-MSA^hyb^	1.005	0.885	2.152	1.196
A1-MSA^hyb^	1.010	0.910	2.805	< 0.1
A2-MSL^hyb^	1.164	1.195	< 0.1	3.450

^a^Average of hybrid isolates with the same genotype

### Origin of sequences in hybrids

**Fig. 3:**
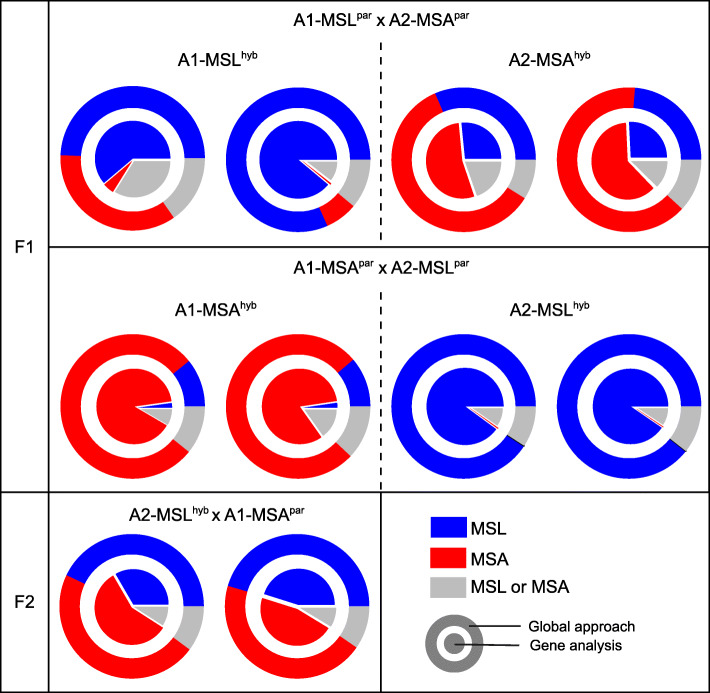
Species’ origin of sequences in F1-hybrids and F2-backcrosses

The “global approach” allowed the assignment of sequence reads from hybrid individuals into one of five paired-origin classes and indicated the occurrence of all pair types in each hybrid. Under *k* = 15, the proportion of reads assigning to “unknown origin” was minimal in most hybrid and backcross genomes and varied from 0.004 to 0.020. Moreover, there was a reasonable proportion of reads in all hybrid genomes that originated undistinguished “from either genome” (0.08–0.16) (Fig. 3). The likely explanation of hybrid reads from types 1 (read derives from MSL) and 2 (read derives from MSA) showed a typical pattern depending on the occurrence of the species-specific mating type chromosome: In hybrids, where the mating type chromosomes derived from MSL parent, a much higher proportion of reads originated from MSL (Average proportion of reads: A1-MSL^hyb^: 0.65; A2-MSL^hyb^: 0.89) than from MSA (Average proportion of reads: A1-MSA^hyb^: 0.21; A2-MSA^hyb^: 0.01). In accordance, hybrids harboring the MSA mating chromosomes showed a higher proportion of MSA reads (average proportion of reads: A1-MSA^hyb^: 0.62; A2-MSA^hyb^: 0.76) than MSL reads (average proportion of reads: A1-MSL^hyb^: 0.28; A2-MSL^hyb^: 0.1), although the species-specific bias was not as strong as in the hybrids with MSL mating type chromosome (Fig. 3). For the F2 backcross genomes, where hybrid individuals have been backcrossed to the MSA parent, the proportions of reads deriving from MSA was slightly higher than in the F1-hybrid genomes. Proportion of MSL reads varied from 0.31 to 0.45 and proportion of MSA reads varied from 0.44 to 0.60 (Fig. 3).

The “gene-dependent” approach, based on the number of inherited genes, indicated similar inheritance patterns similar to the “global approach”. The total number of genes or partial genes that could be detected in the F1-hybrids genomes varied from 5010 to 7206. As above, the ratio of parental genes in a hybrid was dependent on the direction of the cross: for F1-haploid hybrid genomes where the a_1_ mating type chromosomes derived from MSL, a higher number of MSL^par^ genes (average: 4735) than MSA^par^ genes (average: 144) was counted, while for hybrid genomes, where the a_2_ mating type chromosomes derived from MSA, a higher number of MSA genes (average: 4031) than MSL genes (average: 1821) were counted (Fig. 3). The other way around, for F1-haploid hybrid genomes where the a_1_ mating type chromosomes derived from MSA, a higher number of MSA^par^ genes (average: 6007) than MSL^par^ genes (average: 160) were counted, while for hybrid genomes, where the a_2_ mating type chromosomes derived from MSL, a higher number of MSL genes (average: 6459) than MSA genes (average: 24) were counted (Fig. 3). In total, the F1-hybrids harbored more genes inherited by the MSL parent (26347) than genes inherited by the MSA parent (20374), which did not differ significantly (t = 0.54, *p* = 0.3). F1-hybrid-produced haploid genomes of the genotypes A1-MSA^hyb^ showed the lowest number of MSL genes (Fig. 3). The numbers of genes that were similar to both parents and thus could not be tracked varied from 593 to 1697.

For the two genomes of the F2-backcrosses, both genomes showed a lower proportion of MSA genes than the 0.75 expected assuming random recombination and no selection. One F2 isolate contained 2294 MSL genes and 3957 MSA genes (0.37 vs. 0.63), while the other F2 isolate contained 3177 MSL genes and 3251 MSA genes (0.49 vs. 0.51). We identified 211 genes deriving from MSL that were present in all viable F1-hybrid genomes (genotypes A1-MSL^hyb^, A2-MSA^hyb^, A2-MSL^hyb^) and the F2-backcross genomes.

Although there was a tendency that F1-hybrids with higher MSL gene content showed higher infection ability than hybrids with lower MSL gene content, correlation was not significant (r = 0.75; *p* = 0.25) (Additional file 1).

### Genome structure of hybrid genomes and mitochondrial inheritance (Fig. 4)

**Fig. 4:**
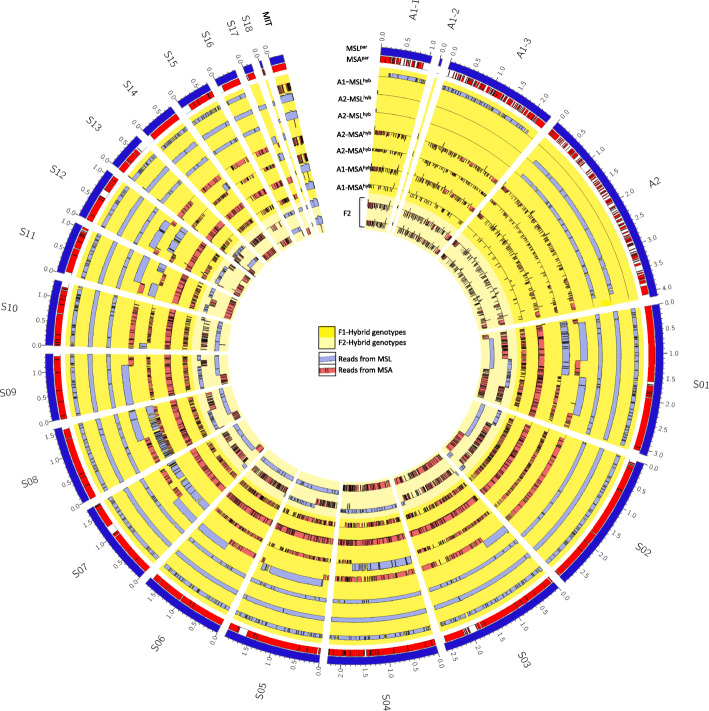
Coverage depth of hybrid genomes. Histograms represent coverage depth of hybrid genomes that are aligned against the the *M. lychnidis-dioicae* (MSL) parent genome (outer blue circle) and the *M. silenes-acaulis* (MSA) parent genome (outer red circle). Blue histograms represent coverage depth for reads deriving from the MSL parent and red histograms represent coverage depth for reads deriving from the MSA parent. F2 hybrid genotypes (outside to inside): A2-MSL^hyb^ × A1-MSA^par^, A2-MSL^hyb^ × A1-MSA^par^, A1-MSL^hyb^ × A2-MSA^par^, A2-MSA^hyb^ × A1-MSA^par^

For all F1-hybrids and two F2-backcrosses, most reads could be assigned to one of the parental species and aligned to the 21 reference scaffolds. However, for one of the eight F1-hybrids (genotype: A1-MSL^hyb^) coverage depth was too low to visualize it along the scaffolds, and thus it was excluded for visualization. Considering the coverage depth of alignments to both parental species at a certain locus, the visualization allows the estimation of the species origin of chromosomes/ scaffolds and chromosomal segment in the hybrid genomes (Fig. 4).

Inheritance of chromosomes or major pieces of chromosomes - visualized by the histograms - showed a clear pattern depending on the origin of the mating type chromosome: For the three F1-hybrid genomes, which harbour the mating type chromosome from MSL (A1-MSL^hyb^ or A2-MSL^hyb^), nearly all chromosomes were inherited from the MSL parent. A similar pattern became visible for the two hybrid genomes where the a_1_ mating type chromosome derives from the MSA parent (A1-MSA^hyb^) parent: nearly all chromosomes were inherited from the MSA parent. Interestingly, in the two F1-hybrid genomes where the a_2_ mating type chromosome derives from MSA (A2-MSA^hyb^), it seemed that recombination occurred: although a majority of chromosomes were inherited by the MSA parent, there were some full chromosomes or chromosome arms inherited by the MSL parent. Additionally, those genomes exhibited the results of recombination within single chromosomes, where some pieces (>1Mbp) derived from the MSL parent and some pieces from the MSA parent. Moreover, it seemed that for some chromosomes (e.g., S08) both parental chromosomes are present in the hybrid genomes, suggesting the occurrence of aneuploidy (Fig. 4). For the two haploid F2 isolates of the genotype A2-MSL^hyb^ × A1-MSA^par^ a higher degree of recombination could be seen, where chromosomes/ chromosome pieces derived either from MSL or MSA.

In contrast to the inheritance patterns described above, the origin of mitochondrial sequences showed a different trend: The hybrid genotypes A1-MSL^hyb^ and A2-MSA^hyb^ that derive from the cross A1-MSL^par^ × A2-MSA^par^ harbour the mitochondrial sequences from the MSA parent, while the hybrid genotypes A1-MSA^hyb^ and A2-MSL^hyb^ that derive from the cross A1-MSA^par^ × A2-MSL^par^ harbour the mitochondrial sequences from the MSL parent. Thus, it seems that mitochondrial sequences were inherited by some control mechanism dependent on the a_2_ mating type chromosome.

### Host adaptation and candidate genes involved in specialization

**Fig. 5:**
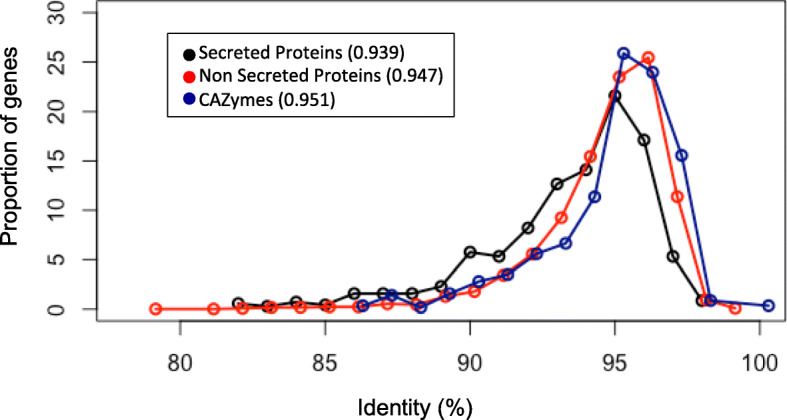
Distribution of nucleotide identities of all protein-encoding genes occurring in both species’ genomes

From 279 genes coding for small secreted proteins (SSP) in the reference genome from the reference *M. lychnidis-dioiceae* genome Lamole p1A1, we found 278 in the MSL genomes and 273 in the MSA genomes, exhibiting a lower average between-species identity than non-secreted proteins (SSP: 0.93, non SSP: 0.94) (Fig. 5). There was no significant difference in the distribution of those genes, indicating no dissimilarity (Kolmogorov-Smirnov-Test, *P*-value = 0.94). For the 236 annotated CAZymes we found 236 in the MSL genomes but only 231 were detected in the MSA genomes, exhibiting a slightly higher similarity (CAZymes: 0.95, complete set of genes: 0.94) than average between-species identity than the complete set of genes (Fig. 5).

Counts of genes from selected PFAM groups in both species’ genomes indicated candidate genes involved in host specificity. Results showed that for most PFAM groups, copy numbers of genes for MSL and MSA were identical, and only three families exhibited different numbers of genes: A Secretory lipase (PF03583.7:) (Number of genes in MSL: 7, Number of genes in MSA: 6), Major Facilitator Superfamily (PF07690.9) (Number of genes in MSL: 119, Number of genes in MSA: 115) and Zinc knuckle (PF00098.16) (Number of genes in MSL: 7, Number of genes in MSA: 5) (Table 3).

**Table 3:** Count genes occurring in parental genomes, F1-hybrid and F2-backcross genomes

	MSL^par^	MSA^par^	A1-MSL^hyb^	A2-MSA^hyb^	A2-MSA^hyb^	A1-MSA^hyb^	A1-MSA^hyb^	A2-MSL^hyb^	A2-MSL^hyb^	A2-MSL^bc^	A2-MSL^bc^
Secreted Proteins	278^a^	273	278^a^ (248^b^/1^c^/29^d^)	275 (75/145/55)	276 (65/160/51)	276 (2/242/32)	269 (3/221/45)	278 (244/0/34)	278 (248/0/30)	276 (92/141/43)	275 (124/121/30)
CAZymes	236	231	236 (226/0/10)	235 (76/124/35)	233 (64/146/23)	235 (3/219/13)	229 (2/214/5)	235 (227/0/8)	235 (227/0/8)	235 (84/128/23)	234 (127/95/12)
Sugar-Transporter	64	63	64 (63/0/1)	63 (17/38/8)	64 (22/38/4)	63 (0/62/1)	63 (0/61/2)	64 (62/0/2)	64 (63/0/1)	64 (20/41/23)	64 (32/31/3)
**PFAM**											
PF00067.15 Cytochrome P450	10	10	10 (10/0/0)	10 (3/6/1)	10 (2/8/0)	10 (0/10/0)	10 (0/10/0)	10 (10/0/0)	10 (10/0/0)	10 (6/4/0)	10 (8/2/0)
PF01697.20 Glycosyltransferase family	5	5	5 (5/0/0)	5 (1/4/0)	5 (1/4/0)	5 (0/5/0)	5 (0/5/0)	5 (5/0/0)	5 (4/0/1)	5 (2/2/1)	5 (4/1/0)
PF06280.5 Fn3-like (DUF1034)	10	10	10 (9/0/1)	10 (3/7/0)	10 (2/7/1)	10 (0/9/1)	10 (0/10/0)	10 (9/0/1)	10 (9/0/1)	10 (2/5/3)	10 (7/2/1)
PF00646.26 F-box	7	7	7 (7/0/0)	7 (3/3/1)	7 (1/5/1)	7 (0/7/0)	7 (0/7/0)	7 (6/0/1)	7 (7/0/0)	7 (1/4/2)	7 (4/2/1)
PF03583.7 Secretory lipase	7	6	7 (6//01)	7 (1/4/2)	7 (6/0/1)	7 (1/4/2)	6 (1/4/1)	7 (6/0/1)	7 (6/0/1)	7 (6/0/1)	7 (6/0/1)
PF07690.9 MFS1 Major Facilitator Superfamily	119	115	118 (114/0/4)	117 (33/64/20)	118 (37/73/8)	115 (0/111/4)	115 (1/109/5)	116 (109/0/7)	116 (113/0/3)	116 (34/73/9)	116 (55/55/6)
PF01753.11 zf-MYND finger	7	7	7 (7/0/0)	7 (3/4/0)	7 (0/7/0)	7 (0/7/0)	7 (0/7/0)	7 (7/0/0)	7 (7/0/0)	7 (0/7/0)	7 (1/6/0)
PF00097.18 Zinc finger, C3HC4 type	14	14	14 (14/0/0)	14 (2/12/0)	14 (4/10/0)	14 (0/14/0)	14 (0/14/0)	14 (14/0/0)	14 (14/0/0)	14 (3/10/1)	14 (5/9/0)
PF00098.16 Zinc knuckle	7	5	7 (7/0/0)	7 (3/3/1)	6 (0/4/2)	6 (1/5/0)	6 (1/5/0)	7 (7/0/0)	7 (7/0/0)	7 (4/3/0)	7 (6/0/1)
PF07738.6 Sad1/UNC-like	2	2	2 (0/0/2)	2 (0/0/2)	2 (0/0/2)	2 (0/0/2)	2 (0/0/2)	2 (0/0/2)	2 (0/0/2)	2 (2/0)	2 (0/2)

^a^ Total count of genes; ^b^ Count of genes originating from the MSL parent; ^c^ Count of genes originating from the MSA parent; ^d^ Count of genes with “unknown origin”

From 211 MSL genes that were consistently present in the viable F1-hybrid genomes (Additional file 2), 10 genes (proportion 0.047) corresponded to the group of secreted proteins, which was slightly higher than the proportion of secreted proteins among all genes in the reference genome. Most of them were with unknown function. For two genes (MVLG_00826 and MVLG_00897) PHI results emphasized best BLAST hits for a chitinase from *Trichoderma* (CHT42) (Identity: 25.87 and 32.17; evalue: 6E-13 and 2E-13). Those two genes fall into the group of CAZymes, where we detected 10 genes in total (proportion 0.047). Here, six genes grouped to the class of Glycosyl Hydrolase (GH), two genes to the class of carbohydrate esterase (CE) and two genes to the class gycosyl transferase (GT). Beside the chitinase hits, one gene of the class of Glycosyl Hydrolase (MVLG_06984) showed a blast hit for the *Ustilago maydis* gene (um00446) (Identitity: 57.75; evalue: 6E-23), which is still uncharacterized.

Regarding the genes from selected PFAM groups, we detected three genes that were present in all F2-hybrid genomes. One gene encoded for a secretory lipase (MVLG_7229), and displays the lipase that was not present in the MSA genome. The two other genes belonged to the Major Facilitator Superfamily (MVLG_00763 and MVLG_06941). PHI results showed that one of them (MVLG_06941) corresponded to the sucrose transporter Srt1 in *Ustilgao maydis* (Identity: 26.22; evalue: 4E-16) and a transporter Hxs1 in *Cryptococcus* (Identity: 36.68; evalue:1E-54), while the best BLAST hit for the second gene corresponded to a transmembrane transporter of *Giberella* (GzMyb019) (Identity: 22.22; evalue:1E-05).

## Discussion

In the present study, we combine infection experiments of host-specific pathogen species and their hybrid offspring, with a focus on their genomic content, providing a better understanding of hybridization and the genetics of host adaptation in plant-fungus interactions.

### Infection ability of hybrids and their genomic composition

In order to gain insights into genetic differences between sibling pathogen species and to identify genes relevant for host specificity, we investigated fungal hybrids by successful crossing of two distinct fungal species *Microbotryum lychnidis-dioicae* (MSL) and *M. silenes-acauli* (MSA) naturally occurring only on their host species *Silene latifola* and *S. acaulis*, respectively. The sequenced hybrid strains represent haploid offspring of teliospores after meiosis and germination. Infection experiments on the host species *S. latifolia* supported a high degree of specialization of these two pathogens, since none of these host plants became infected by *M. silenes-acaulis*, while *M. lychnidis-dioicae* successfully infected the host at high rates. Furthermore, the generated hybrids showed the ability to infect the *S. latifolia* host. The hybrid infection rate was lower in comparison to the parental species that is adapted to the host (MSL), but higher than the non-adapted species (MSA). These results are supported by previous studies with hybrids between different *Microbotryum* species [24, 25]. A lower infection ability can be due to *extrinsic* factors (e.g., lack of adaptation to the host environment) or *intrinsic* factors (e.g., intra-genomic conflicts which occur by combining the set of genes of the two species) that decrease the fitness of hybrids [26, 27].

Consistent with host specialization being strongly determined by genetic traits in this system, the genome analyses of all haploids derived from F1-hybrids revealed a higher number of genes deriving from the fungal species adapted to the host than genes deriving from the non-adapted fungus, supporting the idea that MSL-specific genes contribute to a successful infection. Moreover, F1-hybrids with a higher MSL gene content were more infectious than those with lower MSL content. These results reveal that infection of the host *S. latifolia* is facilitated by a higher proportion of species-specific *M. lychnidis-dioicae* genes and their appropriate adaptations to the host and that extrinsic factors play an important role in the maintenance of distinction fungal lineages within the *Microbotryum* complex [26, 27].

Even though extrinsic factors involving the pathogenic specificities likely influences the genic content of hybrid lineages, multiple insights were also available on the intrinsic incompatibilities between genomes from the fungal species. Sequence analyses and karyotypes showed a similar genome size in the viable F1-hybrid isolates compared to the parental isolates, which might indicate that homoploid hybrid gametes are more viable than aneuploidy and thus contribute either to continuation of the homoploid hybrid lineage or facilitate backcrossing to the parental lineages. Examples of homoploid hybrid speciation have been shown for some plant species, e.g., [28].

Inheritance patterns of species-specific genes or chromosomes did not follow the 50/50 ratio as it would be expected in meiotic products of the F1-hybrids. Especially, hybrid progeny that harbours the MSL mating type chromosome (A1-MSL^hyb^/A2-MSL^hyb^) closely resembles the parental genomes. It seems that meiotic products were most viable if the majority of loci were inherited from a single species. Especially, if we consider, that only successfully mated hybrids and backcrosses are able to infect host plants and therefore the bias towards functional genome organization is extremely high in this experiment. This illustrates the strong influence of intra-genomic incompatibilities in F1-hybrids, e.g., genomic incompatibilities between interacting alleles, Dobzhansky-Muller interactions [29] or negative epistasis effects, as has been reported for yeast hybrids [30].

An alternative hypothesis would be that these F1 progeny were not the result of hybridization and meiosis but originated from an asexual budding event of the mated product, as it has been reported for *Cryptococcus neoformans* [31]. However, this possibility is very unlikely, as we would have a dikaryon containing the nuclei from MSL, thus a solopathogenic strain that can produce teliospores in the host’s anther. Solopathogenic strains have been previously reported for *Ustilago maydis* [32], but to our knowledge, this has not been described for *Microbotryum*.

### Exchange of genomic contents and speciation

Although hybridization occurs in nature [33], including in this fungal system [1], genetics underlying the role of hybrids in speciation or introgression are incompletely understood. Genome analyses of the experimental *Microbotryum* hybrids showed a lack of recombination in three of four post-meiotic genotypes of hybrid genomes, supporting the importance of genetic homogeneity for viable hybrids in *Microbotryum,* as discussed above. Additionally, the mating type chromosomes seem to play a crucial role in intra-genomic compatibility. Depending on the species of origin, the mating type chromosome seems to be the strongest determinant of the amount of inherited species-specific genetic information. Such an effect of mating type chromosomes and linked genes thus might play a central role for hybridization and also speciation in *Microbotryum*. In the hybrid genotypes with a_2_ mating type chromosomes derived from MSA, recombination events (i.e. exchange of genes within a chromosome) were detected, supporting the idea of potential large gene exchanges during hybridization events. Additionally, the data show a chromosome duplication (Contig S08 in isolate C1), revealing this as a possible mechanism for the expansion of genetic regions. Although past events of hybridization and introgressive backcrossing have been detected in the *Microbotryum* complex [1], this is the first time genetic exchange is shown in experimental hybrids. There are different studies using whole genome approaches to detect events of recombination in relation to adaptive traits. For example, it seems that in the plant pathogen *Zymoseptoria tritici*, genes contributing to higher rates of adaptive evolution are located in regions of high recombination [13]. In this context, we hypothesize that the studying of further experimental hybrids in combination with recombination analyses or patterns of genes that are associated with adaptation could be a strategy to identify introgressions of new genes that are relevant for the infection of new hosts.

The genomic analyses of F1-hybrids allowed us to track inheritance patterns of mitochondrial sequences. In the hybrids mitochondrial sequences were not inherited randomly, since all F1- and F2-hybrids contained the mitochondrial genes from the parent with the a_2_-mating type. This uniparental mitochondrial inheritance is known for *Microbotryum* [34] and other basidiomycetes. In *Cryptococcus* and *Ustilago* species, the mitochondrial uniparental inheritance is shown to be controlled by the a_2_ (or MATa) mating type [35, 36], consistent with our findings. This suggests conserved molecular mechanisms over mitochondrial inheritance across Basidiomycota. However, the molecular mechanisms of mitochondrial inheritance and its relevance for hybrid individuals remains to be determined.

### Genetics of host-adaptation and host-specificity candidates

The potential for hybridization or the introgression of genes to generate new genetic diversity strictly depends on the resulting phenotype of the offspring. Especially in plant-pathogenic fungi, there is growing interest in understanding the genetic basis of host adaptation and specificity. Genome comparison of the two species *M. silenes-acualis* and *M. lychnidis-dioicae* revealed that most gene families are shared and the majority of genes are conserved over 95% identity at the sequence level (Fig. 3), indicating that both species possess very similar biological features. Genes encoding small secreted proteins (SSPs) are known to be under diversifying selection, indicating their important role for specialization to the host, as is known from other biotrophic pathogens [5]. In our study, the proportion of those genes were similar in the genomes of both species but showed slightly lower nucleotide identity than the complete genome set. Beckerson et al. 2019 also found SSPs to be differentiated among *Microbotryum* species and also more often under positive selection.

Furthermore it is assumed that the expansion and deletion of certain gene families can be relevant indicators of species-specific interactions [37, 38]. From 7285 detected genes in the MSL genome, we found 7132 orthologs in the MSA genome. Additionally, the similarity between genes of the two (0.94) is remarkably high. Regarding genes from selected PFAM domains that appeared to be expanded in *M. lychdnidis-diocae* [22], we identified one secreted lipase in our study (MVLG_7229) that occurred in both *M. lychnidis-dioacae* parents and was absent in the *M. silenes-acaulis* parents, however it was detected in all viable F1 and F2-hybrid genomes. It is known that in plant-pathogenic fungi, lipases can promote propagule adhesion and plant tissue penetration [39]. Moreover, lipases can be involved in nutrient uptake from the host or in the inhibition of immunity-related callose formation [40]. Similarly, two genes of the Major Facilitator Superfamily domain family were missing in the *M. silenes-acaulis* genomes, but were present in all F2 genomes. Genes from this family often encode for membrane proteins and are involved in transmembrane transport processes [41]. Comparison to the PHI-base revealed similarity to the sugar transporter Srt1 in *Ustilago maydis* and Frt1 in *Botrytis* indicating substrate-specific transport in *M. lychnidis-diocae*. These findings are also supported by the results of the recent genome analyses by [42]: they found facility transporters and a secreted lipase in *Microbotryum* to be under positive selection, reinforcing the idea that selection upon those genes in the host-species-driven selection is a reflections of their involvement in host adaptation.

So far, none of the above mentioned genes have been identified specifically as genes mediating host-specificity in prior studies, but our approach using experimental backcrossed hybrids indicates that some of them might be relevant for infecting *S. latifolia* or at least their specific regulation during the infection process is relevant for successful sporulation on the respective host (Fig. 1). By the application of experimental selection, we identified a set of 211 *Microbotryum lychnidis-dioceae* genes that were present in infectious F1-hybrids and haploid F2-backcrosses (Additional file 2). Within those genes the proportion of genes encoding for secreted proteins and CAZymes was higher than in the entire genome. Secreted CAZymes are required for plant infection. Most of the detected CAZymes belonged to the GH18 family, known for breaking glycosidic bonds in the plant wall [43] [22]. showed that *M. lychnidis- dioicae* is lacking xylanases, which might indicate the high relevance of glycosyl hydrolases for cell wall degradation [37]. showed that these hydrolases are highly upregulated during infection in *Melampsora* and *Puccinia*.

In conclusion, the limited number of hybrids, that were analyzed, does not allow to directly infer genotype-phenotype relations. However, the discussed genes represent an initial set of candidate genes responsible for specialization that will have to be tested in further studies. For example, the effect of those candidate genes during infection could be examined by knockout mutants.

## Conclusion

The use of experimental fungal hybrids in combination with infection assays and genome analyses is a promising way to investigate the evolution of hybridization and host specialization in pathogenic interactions. The study demonstrates the importance of intra-genomic compatibility in hybrids and the high influence of the species-specific mating type chromosomes in fungal hybrids. Additionally, experimental selection and the analyses of selected functional groups reveal the importance of small secreted proteins for host-specific interaction and provide a subset for candidate genes involved in host-specificity, that present a valuable base of further investigations.

## Methods

### Studied species

The two *Microbotryum* species, *M. lychnidis-dioicae* (MSL) and *M. silenes-acaulis* (MSA), belong to the group of the anther-smut fungi on the Caryophyllaceae, occurring on the host species *Silene latifolia* and *Silene acaulis*, respectively. The current study uses the MSL strain “Lamole” ([22, 44]) and the MSA strain from [45]. The two hosts are perennial species that are adapted to different ecological niches. While *S. latifolia* is a short-lived perennial weed that grows in open disturbed habitats, *S. acaulis* is a very long-lived perennial that is restricted to arctic-alpine environments in the northern hemisphere ([46]).

### Host specificity of parental Microbotryum strains

Viability and host specificity of the two parental *Microbotryum* species, *M. lychnidis-dioiceae* and *M. silenes-acaulis*, were estimated by infection ability following experimental inoculation on the host *S. latifolia*. Seeds of the host species *S. latifolia* (Hadley Population, Massachusetts (2012), 42.34339–72.612127) were collected by Michael M. Hood according to national and international legislation. Especially, all sampling material and experiments were complying to the rules of the Convention on the Trade in Endangered Species of Wild Fauna and Flora. Seed were surface-sterilized in a solution containing 10% bleach, 50% ethanol and 40% sterile water and germinated at 24 °C on 0.8% agar with 0.1 × MS salts [47].

For the inoculum, pairs of haploid isolates of a_1_ and a_2_ mating types from the same meiosis were prepared for both species (by the germination of the field-collected teliospores on potato dextrose agar (PDA; Difco), followed by isolation of meiotic tetrads using micromanipulation, as reported by [48]. Mating types of the haploid, yeast-like cultures were determined by PCR amplification of the pheromone receptor gene, as by [49]. For intraspecific mating and inoculation, each culture was suspended at 4 × 10^7^ cells/mL on sterile deionized water, and suspensions of opposite mating type were mixed in equal amounts. Inoculum was applied to 50 *S. latifolia* plants per fungal species by pipetting 4 μL of the cell suspension onto the apical meristem of 10-days-old seedlings and incubating at 15 °C for 2 days. Afterwards seedlings were transplanted to soil and grown under greenhouse conditions [50]. When plants flowered, the number of healthy and diseased plants (i.e. flowers with spore-filled anthers) was recorded.

### Production of F1-hybrids

Analyses were based on inoculation of host plants with experimental hybrids between the two fungal species, *M. lychnidis-dioicae* and *M. silenes-acaulis*, in reciprocal combinations that allowed us to separately assess the effect of the mating type chromosomes. With regard to the mating type locus, two combinations of *Microbotryum* F1-hybrids were generated: F1-hybrids with the a_1_ mating type from *M. lychnidis-dioicae* and the a_2_ mating type from *M. silenes-acaulis* (referred to as A1-MSL^par^ × A2-MSA^par^; “par” indicating parental species) and the reciprocal F1-hybrids (referred to as A1-MSA^par^ × A2-MSL^par^) (Fig. 1). For the interspecific inoculum, the four haploid parental isolates were used as described above, with mixtures of cell suspensions from opposite mating type from different species. For each crossing-combination 4 μl of inoculum were applied to 50 plants following the procedure described above.

### Production of F2-backcrosses

To enrich for host-specificity loci, selection was imposed by the particular host species environment upon F2-backcrossed genotypes. Haploid gametes derived from the F1-hybrids were backcrossed to the original haploid parental strains of *M. silenes-acaulis* (MSA) and again inoculated onto the host *S. latifolia*. Under the expectation of free recombination, these genome combinations were expected to be approximately ¾ MSA and ¼ MSL, while the selective environment was the native host of MSL. Regarding the possible origins of the mating types (e.g., A1-MSL^hyb^, A2-MSL^hyb^, A1-MSA^hyb^, A2-MSA^hyb^, where the species abbreviation indicates the parental origin of the mating type chromosome and “hyb” indicates hybrids derived from F2-backcrosses), this approach produced F2-backcrosses combinations where both mating types were derived from MSA (homospecific mating type backcross) or where the mating type derives from both parents (heterospecific mating type backcross) (Fig. 1).

In preparation for the production of backcrossed hybrids, spores for each F1-hybrid diploid genotype (A1-MSL^par^ × A2-MSA^par^ and A1-MSA^par^ × A2-MSL^par^) were spread on PDA for 2 days at room temperature. Haploid cells derived from meiosis of the F1-hybrids were isolated via a dilution series, as by [25]. After 2 weeks of growth, mating types of haploid cultures derived from the F1-hybrids were determined by mating type tests as by [51] with the MSL parents as tester strains. Only those cultures where mating type was confirmed by conjugation were used for further analyses (Fig. 1).

For each of the four haploid gamete types derived from the F1-hybrids (A1-MSL^hyb^, A2-MSA^hyb^, A1-MSA^hyb^, A2-MSL^hyb^_;_ indicating the parental species of origin for the mating type chromosomes) at least 30 haploid cultures of each mating type were obtained and used for backcrossing. Depending on the number of cultures generated in the F1-hybrids, isolates with the same mating type were pooled and backcrossed to the MSA parental haploid culture of the opposite mating type (Fig. 1). Haploid cells of each isolate were suspended in sterile deionized water, isolates pooled and concentration adjusted to equal the concentration of the parental MSA isolate of opposite mating type (Additional file 3). F1-hybrid-produced-haploid cultures and parental MSA culture were mixed and used for the inoculation of 750 *S. latifolia* seedlings as described above. After flowering and successful infection, diploid F2 spores were collected. In order to obtain haploid isolates from the F2-backcross pathogens for genomic analyses, spores were germinated on PDA, and haploid cultures were obtained via dilution steps as indicated above [25].

### Genome sequencing, mapping and assembly

For the parental haploid isolates from each fungal species, genomic DNA was isolated from yeast cultures by the method of [52]. Paired-end libraries were constructed using Nextera technology, according to the manufacturer’s instructions, and sequencing was performed using the Illumina MiSeq platform chemistry at the Genomics Service Unit of the Ludwig-Maximilian-University Munich, Biocenter. Reads were pre-processed using trimmomatic [53] and fastx [54]. First, adapter sequences were trimmed, and reads with more than 25% low-quality nucleotides with a Phred quality score < 30 were discarded. Reads shorter than 75 BP were discarded.

Reads from all four parental haploid genomes were mapped to the nucleic reference genome (*Lamole p1A1* [22]) using the burrows-wheel-aligner (bwa) with default settings and aligned reads filtered using bam2fastq [55, 56]. For the *M. lychnidis-dioicae* genomes, mapping alignments were used as target for the assembly with velvet columbus 1.2.20 with k-mer values from 21 to 79 [57]. The M*. silenes-acaulis* genomes were assembled de novo using velvet with k-mer values from 21 to 79. Assembly quality and characteristics was observed using quast 2.3 [58].

Concerning the F1-hybrid-generation we sequenced eight haploid isolates (two isolates for each of the four genotypes A1-MSL^hyb^, A2-MSA^hyb^, A1-MSA^hyb^, A2-MSL^hyb^). From the F2-backcrosses we used two haploid isolates of the backcross type A2-MSL^hyb^ × A1-MSA^par^ for genomic analyses. Thus, genomic DNA from eight haploid F1-hybrids and two F2-hybrid strains was isolated, sequenced and pre-processed as indicated above.

### Gene content and sequence divergence of parental genomes

To identify genes in the newly assembled MSL and MSA genomes, 7364 genes from the reference genome Lamole p1A1, were aligned to the MSL and MSA genomes using mummer 3.2.3 with the nucmer algorithm [59]. Contigs that corresponded to a reference gene sequence (> 80% identity) were extracted and trimmed according to the gene size using bedtools2.25.0 with the getfasta implement [60]. Occurrence of (partial) genes in parental genomes was counted.

### Genetic distance of F1-hybrid genomes to parental genomes by electrophoretic karyotypes

We first estimated genetic distance of eight F1-hybrid individuals to parental genomes based on similarities of electrophoretic karyotypes. Therefore, each of the four parental and F1-hybrid isolates was subjected to pulsed field gel electrophoresis as by [61]. Two gels were run with a maximum of 10 samples, including all four parental samples in both gels. Gel images were analyzed using the software ImageJ [62]. A region of the image between 0.5 and 3.0 Mbp was selected across the lanes and an electropherogram (densitometry plot) was calculated. Baseline and background were subtracted, and the peak height of a single chromosome band within each karyotype was determined and used to standardize variation in brightness across lanes. Pairwise comparisons to both parental species were conducted by summing the squared differences in height at each pixel along the length of the paired electropherogram as in [61]. Values of genetic distance were standardized among gels by division by the genetic distance of parental genomes of each gel. Thus, a quotient lower than 1 indicated greater similarity than both parents to each other while a quotient higher than 1 indicated the degree of dissimilarity.

### Sequencing of hybrid genomes and genetic distance to parental genomes

We estimated the genetic distance by the occurrence of single-nucleotide polymorphisms in hybrid reads. Hybrid reads were aligned to the gene sequences of both original parental genomes using bwa with default settings. Alignments with variants (including single-nucleotide-polymorphisms, insertions, deletions) were determined and filtered using vcftools with the samtools mpileup and the bcftools view implements [55, 63] and only alignments with coverage between 5 and 100 were captured. For all alignments, the number of variants was counted and overall nucleotide identity calculated.

### Origin of sequences in hybrid and backcross genomes

We applied two approaches to track from which parental genomes alleles had been inherited by the hybrids. The first approach is based on a gene-independent analyses without any need of assembling the parental genomes or annotating genes. Therefore each single read is classified into one of the following categories i) derives from MSL parent, ii) derives from MSA parent, iii) derivation unknown (Additional file 5).

The second approach is based on the alignment of hybrid reads to the reference genes and the observation of variants. Therefore, gene sequences in parental genomes were used as target and hybrid reads were aligned to the gene sequences of both original parental genomes using bwa with default settings and variants determined via vcftools as indicated above. For all alignments, coverage distribution was observed and alignments with coverage too low or too high from the expected coverage were discarded. Next, we counted the number of genes that were hit and not hit in the reference gene list for both parental genomes. For each hybrid, genes were categorized into three classes; a) no alignment - if no hybrid read hit the parent gene, b) alignment without variants and c) alignment with variants. To make a decision about which parental gene is the likely original sequence, categorized gene lists from both parents were compared, and each of the hybrid 7364 genes was classified into five categories: 1) Genes, that are identical to both parents (no variants should exist in either alignment), 2) genes from MSL parent (variants in alignment for MSA but not for MSL), 3) genes from MSA parent (variants in alignment for MSL but not for MSA), 4) genes with variants to both parents, and 5) genes that were not present in the hybrids. Following this strategy, we counted MSL genes that were present in all F1 genomes and F2 genomes, respectively.

To check for a correlation between genomic content of F1-hybrids and their infection ability, we computed species-specific gene content of each hybrid genotype and tested for significant correlation (*p*-value threshold 0.05) with the infection ability in the F2-generation using *Pearson’s correlation coefficient.*

### Genome structure of hybrid genomes and mitochondrial inheritance

In order to visualize the structure of the hybrid genomes, the MSL genome assembly from [21] which is composed of 23 contigs including the two mating type chromosomes a_1_ and a_2_ (contigs: A1–1, A1–2, A1–3, A2) was used as reference genome assembly. Therefore, reads from the eight F1 and two F2-hybrid genomes, were aligned to the MSL genome [21] and the MSA genome (assembly from this study) using bwa with standard parameters. For each read, genetic distance to both parents was determined from SAM files using samtools [64]. Afterwards, distance to both parents was compared and read assigned to the parent with smaller genetic distance. Next, for each position in the “original” reference, coverage depth was in reported 10 kb windows, and histograms were visualized via circos0.69 [65].

With regards to inheritance patterns of mitochondrial sequences, we also aligned hybrid reads against the mitochondrial sequences from MSL [22] and MSA (this study), reported coverage depth in 1 kb windows and included it in the visualization.

### Host adaptation and functional assignment

Studies on other phytopathogenic fungi show that genes encoding for secreted proteins and carbohydrate active enzymes (CAZymes), harbor potential virulence genes and factors that interact with the host [5, 43]. Additionally, [22] showed that genes from 8 PFAM-domains, appeared to be expanded in *Microbotryum lychnidis-dioicae* in comparison to other plant-pathogenic fungi. Based on the gene annotation of [22], and with regard to the virulence and host specificity factors, we focused on those groups to detect genes or groups involved in host specialization. Therefore parental gene sequences corresponding to those groups were extracted and similarity between the parental species calculated and visualized via identity plots. Moreover, we observed the occurrence of the mentioned groups of genes in the hybrid genomes.

As the experimental design selects for loci that are responsible for the adaptation to *M. lychnidis-dioicae,* we counted MSL genes that were present in all hybrid or backcross genomes. Function of detected MSL genes was assessed by available annotations of the reference genome by [22]. For those genes where no functional description was available, genes were compared to the PHI-base 3.8 database (Pathogen-Host-Interaction: http://www.phi-base.org) [66] using BLAST, and only hits with an E-value < 10^− 5^ were captured.

## Supplementary information

**Additional file 1. **Diagram (.pdf) representing the infection ability of hybrids in dependence on the genotype. Correlation between proportion of species-specific genes in haploid F1-hybrid genomes and their infection ability after backrossing to *M. silenes-acaulis*.

**Additional file 2. **Text file (.docx) listing the genes that are present in all infectious hybrid genomes. *M. lychndidis-dioicae* genes from the reference genome Lamole p1A1 that are present in all infectious F1-hybrids and F2-backcrosses.

**Additional file 3.** Text file (.docx) listing the used inoculum. Haploid F1-strains used for inoculum.

**Additional file 4.** Gel picture (.pdf) showing the karyotypes of four F1-hybrids and parental strains. Electrophoretic karyotypes *for Microbotryum silenes-dioicae* and *M. silenes-acaulis* and F1-hybrids resulting from crosses between the two species.

**Additional file 5. **Text file (.docx) containing the script for the “global approach”. Detailed script for tracking the origin of reads in hybrids.

**Additional file 6.** Diagram (.pdf) representing coverage histogram for the “global approach”. Example for a coverage histogram from which sequencing coverage *c* and the optimal threshold C* was inferred.

## Data Availability

Pre-processed reads and assemblies that are used for analyses during the current study were deposited in the European Nucleotide Archive database (http://www.ebi.ac.uk/ena, accession number PRJEB36154).

## Abbreviations

MSL: *Microbotryum lychnidis-dioiceae*
MSA: *Microbotryum silenes-acaulis*
A1-MSL^par^/A2-MSL^par^: Haploid parental strains from MSL with mating type a_1_ or a_2_
A1-MSA^par^/A2-MSA^par^: Haploid parental strains from MSA with mating type a_1_ or a_2_
A1-MSL^par^ × A2-MSA^par^: Cross between the parental strains A1-MSL and A2-MSA
A1-MSA^par^ × A2-MSL^par^: Cross between the parental strains A1-MSA and A2-MSL
A1-MSL^hyb^/A2-MSL^hyb^: Haploid hybrid strain that derives from a cross between MSL and MSA where the mating type A1/ A2 derives from A1-MSL^par^/ A2-MSL^par^
A1-MSA^hyb^/A2-MSA^hyb^: Haploid hybrid strain that derives from a cross between MSL and MSA where the mating type A1/ A2 derives from A1-MSA^par^/ A2-MSA^par^
