# Supplementary material for: Meiotic recombination in the offspring of Microbotryum hybrids and its impact on pathogenicity
Source: BMC Evol Biol. 2020 Sep 17;20:123. doi: 10.1186/s12862-020-01689-2 (PMC7499883; doi:10.1186/s12862-020-01689-2)
Supplement: Supplementary file 1 — Additional file 1. Diagram (.pdf) representing the infection ability of hybrids in dependence on the genotype. Correlation between proportion of species-specific genes in haploid F1-hybrid genomes and their infection ability after backrossing to M. silenes-acaulis. [file 12862_2020_1689_MOESM1_ESM.pdf]

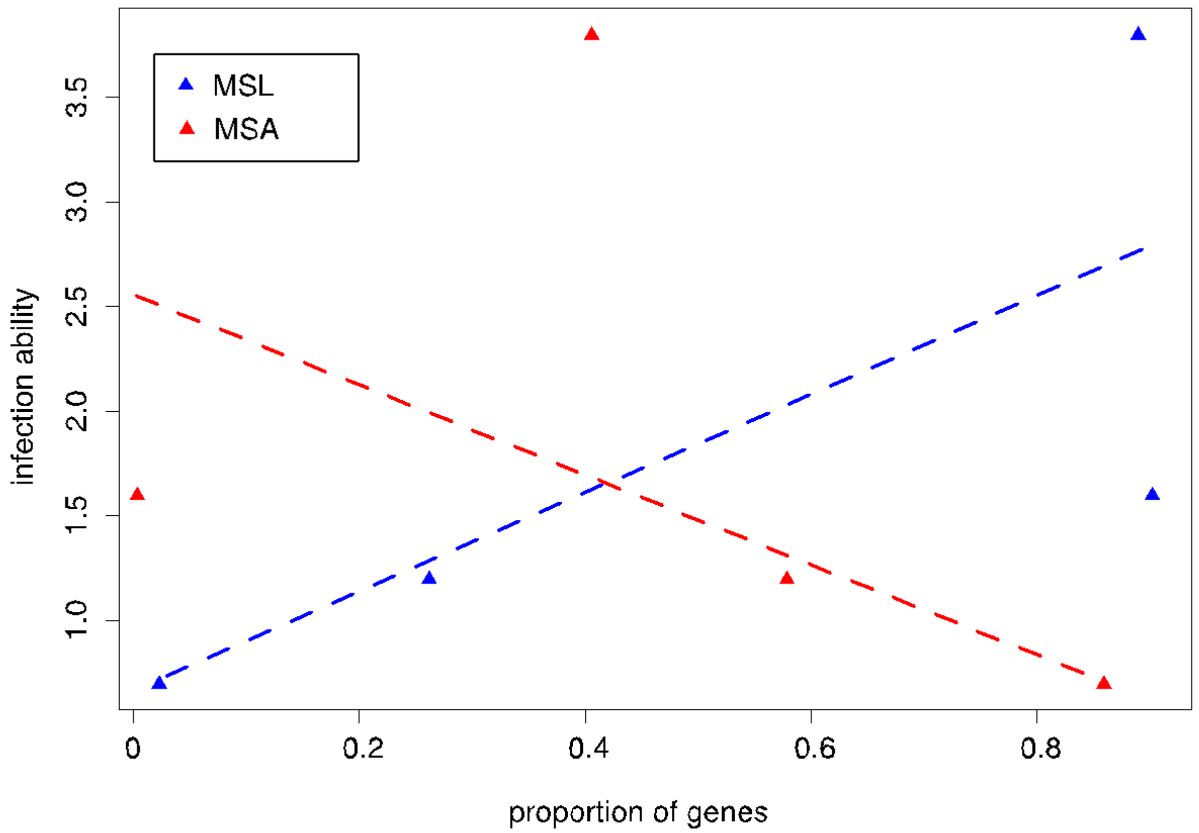

Figure S2: Correlation between proportion of species-specific genes in haploid F1-hybrid genomes and their infection ability after backrossing to *M. silenes-acaulis*.
