# Supplementary material for: Meiotic recombination in the offspring of Microbotryum hybrids and its impact on pathogenicity
Source: BMC Evol Biol. 2020 Sep 17;20:123. doi: 10.1186/s12862-020-01689-2 (PMC7499883; doi:10.1186/s12862-020-01689-2)
Supplement: Supplementary file 2 — Additional file 2. Text file (.docx) listing the genes that are present in all infectious hybrid genomes. M. lychndidis-dioicae genes from the reference genome Lamole p1A1 that are present in all infectious F1-hybrids and F2-backcrosses. [file 12862_2020_1689_MOESM2_ESM.docx]

**Table S2**. *M. lychndidis-dioicae* genes from the reference genome Lamole p1A1 that are present in all infectious F1-hybrids and F2-backcrosses.

| **Region** | **LOCUS** | **NAME** | **ANNOTATION** | | |  |
| --- | --- | --- | --- | --- | --- | --- |
| NRR | MVLG_05354 | hypothetical protein |  |  |  | |
| NRR | MVLG_05911 | hypothetical protein |  |  |  | |
| NRR | MVLG_06237 | hypothetical protein |  |  |  | |
| NRR | MVLG_06652 | hypothetical protein |  |  |  | |
| NRR | MVLG_06735 | hypothetical protein |  |  |  | |
| NRR | MVLG_06984 | hypothetical protein | Pfam | PF00933.14 | Glyco_hydro_3 | |
| NRR | MVLG_07142 | hypothetical protein |  |  |  | |
| NRR | MVLG_07168 | hypothetical protein |  |  |  | |
| NRR | MVLG_07251 | hypothetical protein |  |  |  | |
| NRR | MVLG_07297 | hypothetical protein |  |  |  | |
| NRR | MVLG_07316 | hypothetical protein |  |  |  | |
| PAR | MVLG_03748 | chitin synthase | Pfam | PF03142.8 | Chitin_synth_2 | |
| Autosomal | MVLG_00390 | hypothetical protein |  |  |  | |
| Autosomal | MVLG_00496 | hypothetical protein |  |  |  | |
| Autosomal | MVLG_00680 | hypothetical protein |  |  |  | |
| Autosomal | MVLG_00708 | hypothetical protein | Pfam | PF02558.9 | ApbA | |
| Autosomal | MVLG_00711 | hypothetical protein | Pfam | PF00400.25 | WD40 | |
| Autosomal | MVLG_00712 | hypothetical protein | Pfam | PF00106.18 | adh_short | |
| Autosomal | MVLG_00716 | hypothetical protein |  |  |  | |
| Autosomal | MVLG_00719 | hypothetical protein |  |  |  | |
| Autosomal | MVLG_00720 | uracil phosphoribosyltransferase | Pfam | TIGR01091 | TIGR01091 | |
| Autosomal | MVLG_00721 | hypothetical protein | Pfam | PF00076.15 | RRM_1 | |
| Autosomal | MVLG_00722 | hypothetical protein | Pfam | PF00153.20 | Mito_carr | |
| Autosomal | MVLG_00725 | hypothetical protein | Pfam | PF06414.5 | Zeta_toxin | |
| Autosomal | MVLG_00728 | hypothetical protein | Pfam | PF01694.15 | Rhomboid | |
| Autosomal | MVLG_00729 | hypothetical protein |  |  |  | |
| Autosomal | MVLG_00734 | hypothetical protein | Pfam | PF00887.12 | ACBP | |
| Autosomal | MVLG_00735 | hypothetical protein | Pfam | TIGR03804 | TIGR03804 | |
| Autosomal | MVLG_00736 | hypothetical protein | Pfam | PF02991.9 | MAP1_LC3 | |
| Autosomal | MVLG_00738 | hypothetical protein | Pfam | PF01535.13 | PPR | |
| Autosomal | MVLG_00739 | hypothetical protein |  |  |  | |
| Autosomal | MVLG_00740 | hypothetical protein | Pfam | PF00004.22 | AAA | |
| Autosomal | MVLG_00741 | hypothetical protein |  |  |  | |
| Autosomal | MVLG_00744 | hypothetical protein | Pfam | TIGR00458 | TIGR00458 | |
| Autosomal | MVLG_00750 | hypothetical protein |  |  |  | |
| Autosomal | MVLG_00763 | hypothetical protein | Pfam | PF07690.9 | MFS_1 | |
| Autosomal | MVLG_00767 | hypothetical protein | Pfam | TIGR00981 | TIGR00981 | |
| Autosomal | MVLG_00769 | hypothetical protein | Pfam | PF02146.10 | SIR2 | |
| Autosomal | MVLG_00776 | hypothetical protein | Pfam | PF08325.3 | WLM | |
| Autosomal | MVLG_00777 | hypothetical protein | Pfam | PF10282.2 | Muc_lac_enz | |
| Autosomal | MVLG_00781 | hypothetical protein | Pfam | PF09811.2 | Yae1_N | |
| Autosomal | MVLG_00782 | hypothetical protein | Pfam | PF03587.7 | EMG1 | |
| Autosomal | MVLG_00784 | hypothetical protein |  |  |  | |
| Autosomal | MVLG_00793 | hypothetical protein | Pfam | PF05827.5 | ATP-synt_S1 | |
| Autosomal | MVLG_00798 | hypothetical protein | Pfam | TIGR01464 | TIGR01464 | |
| Autosomal | MVLG_00800 | hypothetical protein | Pfam | PF10187.2 | Nefa_Nip30_N | |
| Autosomal | MVLG_00801 | NADH-ubiquinone oxidoreductase 51 kDa subunit | Pfam | PF10531.2 | SLBB | |
| Autosomal | MVLG_00802 | hypothetical protein | Pfam | PF05730.4 | CFEM | |
| Autosomal | MVLG_00812 | hypothetical protein | Pfam | PF00160.14 | Pro_isomerase | |
| Autosomal | MVLG_00815 | hypothetical protein | Pfam | PF05730.4 | CFEM | |
| Autosomal | MVLG_00817 | hypothetical protein | Pfam | PF05255.4 | UPF0220 | |
| Autosomal | MVLG_00818 | fumarate hydratase | Pfam | PF10415.2 | FumaraseC_C | |
| Autosomal | MVLG_00820 | hypothetical protein |  |  |  | |
| Autosomal | MVLG_00824 | hypothetical protein | Pfam | PF05195.9 | AMP_N | |
| Autosomal | MVLG_00825 | hypothetical protein | Pfam | PF00134.16 | Cyclin_N | |
| Autosomal | MVLG_00826 | hypothetical protein | Pfam | PF00704.21 | Glyco_hydro_18 | |
| Autosomal | MVLG_00827 | splicing factor 3B subunit 1 | Pfam | PF08920.3 | SF3b1 | |
| Autosomal | MVLG_00830 | hypothetical protein |  |  |  | |
| Autosomal | MVLG_00834 | 40S ribosomal protein S28 | Pfam | PF01200.11 | Ribosomal_S28e | |
| Autosomal | MVLG_00835 | hypothetical protein | Pfam | PF04241.8 | DUF423 | |
| Autosomal | MVLG_00841 | glucose 1-dehydrogenase | Pfam | PF00106.18 | adh_short | |
| Autosomal | MVLG_00848 | O-sialoglycoprotein endopeptidase | Pfam | PF00814.18 | Peptidase_M22 | |
| Autosomal | MVLG_00849 | hypothetical protein | Pfam | PF01501.13 | Glyco_transf_8 | |
| Autosomal | MVLG_00887 | hypothetical protein | Pfam | PF00704.21 | Glyco_hydro_18 | |
| Autosomal | MVLG_00919 | hypothetical protein |  |  |  | |
| Autosomal | MVLG_01079 | hypothetical protein | Pfam | PF00505.12 | HMG_box | |
| Autosomal | MVLG_01081 | hypothetical protein | Pfam | PF06283.4 | ThuA | |
| Autosomal | MVLG_01083 | hypothetical protein | Pfam | PF00560.26 | LRR_1 | |
| Autosomal | MVLG_01084 | cell division control protein 3 | Pfam | PF00735.11 | Septin | |
| Autosomal | MVLG_01086 | hypothetical protein | Pfam | PF07653.10 | SH3_2 | |
| Autosomal | MVLG_01087 | hypothetical protein | Pfam | PF08294.4 | TIM21 | |
| Autosomal | MVLG_01088 | hypothetical protein |  |  |  | |
| Autosomal | MVLG_01089 | hypothetical protein |  |  |  | |
| Autosomal | MVLG_01096 | hypothetical protein |  |  |  | |
| Autosomal | MVLG_01097 | hypothetical protein | Pfam | PF03311.7 | Cornichon | |
| Autosomal | MVLG_01099 | isocitrate dehydrogenase [NAD] subunit 1 | Pfam | PF00180.13 | Iso_dh | |
| Autosomal | MVLG_01104 | hypothetical protein | Pfam | PF01988.12 | VIT1 | |
| Autosomal | MVLG_01107 | hypothetical protein | Pfam | PF00857.13 | Isochorismatase | |
| Autosomal | MVLG_01111 | hypothetical protein | Pfam | PF01633.13 | Choline_kinase | |
| Autosomal | MVLG_01114 | hypothetical protein | Pfam | PF01522.14 | Polysacc_deac_1 | |
| Autosomal | MVLG_01117 | hypothetical protein | Pfam | PF00400.25 | WD40 | |
| Autosomal | MVLG_01119 | hypothetical protein |  |  |  | |
| Autosomal | MVLG_01126 | hypothetical protein | Pfam | PF08614.4 | ATG16 | |
| Autosomal | MVLG_01127 | eukaryotic translation initiation factor 2 subunit alpha | Pfam | PF07541.5 | EIF_2_alpha | |
| Autosomal | MVLG_01129 | hypothetical protein | Pfam | PF00271.24 | Helicase_C | |
| Autosomal | MVLG_01131 | cytochrome b-c1 complex subunit Rieske | Pfam | PF00355.19 | Rieske | |
| Autosomal | MVLG_01133 | hypothetical protein | Pfam | PF07992.7 | Pyr_redox_2 | |
| Autosomal | MVLG_01134 | hypothetical protein | Pfam | PF00583.17 | Acetyltransf_1 | |
| Autosomal | MVLG_01135 | hypothetical protein | Pfam | PF05700.4 | BCAS2 | |
| Autosomal | MVLG_01138 | hypothetical protein |  |  |  | |
| Autosomal | MVLG_01142 | hypothetical protein |  |  |  | |
| Autosomal | MVLG_01143 | CMGC/CDK/CDK5 protein kinase | Pfam | PF00069.18 | Pkinase | |
| Autosomal | MVLG_01145 | hypothetical protein | Pfam | PF00067.15 | p450 | |
| Autosomal | MVLG_01147 | hypothetical protein | Pfam | PF06212.5 | GRIM-19 | |
| Autosomal | MVLG_01148 | hypothetical protein |  |  |  | |
| Autosomal | MVLG_01154 | hypothetical protein | Pfam | PF00378.13 | ECH | |
| Autosomal | MVLG_01156 | hypothetical protein | Pfam | PF01593.17 | Amino_oxidase | |
| Autosomal | MVLG_01163 | hypothetical protein | Pfam | PF01062.14 | Bestrophin | |
| Autosomal | MVLG_01167 | hypothetical protein |  |  |  | |
| Autosomal | MVLG_01171 | hypothetical protein | Pfam | PF06423.5 | GWT1 | |
| Autosomal | MVLG_01172 | hypothetical protein | Pfam | TIGR01571 | TIGR01571 | |
| Autosomal | MVLG_01175 | hypothetical protein |  |  |  | |
| Autosomal | MVLG_01177 | hypothetical protein |  |  |  | |
| Autosomal | MVLG_01179 | hypothetical protein | Pfam | PF05773.15 | RWD | |
| Autosomal | MVLG_01182 | hypothetical protein | Pfam | PF01730.9 | UreF | |
| Autosomal | MVLG_01185 | glutaryl-CoA dehydrogenase | Pfam | PF00441.17 | Acyl-CoA_dh_1 | |
| Autosomal | MVLG_01186 | hypothetical protein | Pfam | PF08240.5 | ADH_N | |
| Autosomal | MVLG_01190 | hypothetical protein | Pfam | PF08216.4 | DUF1716 | |
| Autosomal | MVLG_01192 | hypothetical protein |  |  |  | |
| Autosomal | MVLG_01196 | hypothetical protein | Pfam | PF03481.6 | SUA5 | |
| Autosomal | MVLG_01199 | hypothetical protein | Pfam | PF04707.7 | PRELI | |
| Autosomal | MVLG_01201 | C-22 sterol desaturase | Pfam | PF00067.15 | p450 | |
| Autosomal | MVLG_01205 | hypothetical protein | Pfam | PF00620.20 | RhoGAP | |
| Autosomal | MVLG_01255 | hypothetical protein |  |  |  | |
| Autosomal | MVLG_01256 | hypothetical protein |  |  |  | |
| Autosomal | MVLG_01507 | hypothetical protein |  |  |  | |
| Autosomal | MVLG_01795 | hypothetical protein | Pfam | PF04082.11 | Fungal_trans | |
| Autosomal | MVLG_01796 | DNA-directed RNA polymerase II subunit RPB2 | Pfam | PF04563.8 | RNA_pol_Rpb2_1 | |
| Autosomal | MVLG_01797 | hypothetical protein | Pfam | PF05843.7 | Suf | |
| Autosomal | MVLG_01801 | hypothetical protein | Pfam | PF01926.16 | MMR_HSR1 | |
| Autosomal | MVLG_01802 | hypothetical protein | Pfam | PF06862.5 | DUF1253 | |
| Autosomal | MVLG_01803 | hypothetical protein | Pfam | PF02037.20 | SAP | |
| Autosomal | MVLG_01804 | STE/STE11/CDC15 protein kinase | Pfam | PF00069.18 | Pkinase | |
| Autosomal | MVLG_01807 | hypothetical protein |  |  |  | |
| Autosomal | MVLG_01808 | hypothetical protein |  |  |  | |
| Autosomal | MVLG_01809 | hypothetical protein | Pfam | PF01416.13 | PseudoU_synth_1 | |
| Autosomal | MVLG_01812 | hypothetical protein | Pfam | PF00856.21 | SET | |
| Autosomal | MVLG_01813 | hypothetical protein | Pfam | PF09073.3 | BUD22 | |
| Autosomal | MVLG_01814 | glutamate decarboxylase | Pfam | PF00266.12 | Aminotran_5 | |
| Autosomal | MVLG_01820 | hypothetical protein | Pfam | PF07992.7 | Pyr_redox_2 | |
| Autosomal | MVLG_01825 | aspartate aminotransferase | Pfam | PF00155.14 | Aminotran_1_2 | |
| Autosomal | MVLG_01828 | hypothetical protein | Pfam | PF09751.2 | Es2 | |
| Autosomal | MVLG_01829 | hypothetical protein | Pfam | PF00249.24 | Myb_DNA-binding | |
| Autosomal | MVLG_01830 | hypothetical protein | Pfam | PF00005.20 | ABC_tran | |
| Autosomal | MVLG_01875 | hypothetical protein | Pfam | PF11917.1 | DUF3435 | |
| Autosomal | MVLG_01957 | hypothetical protein |  |  |  | |
| Autosomal | MVLG_01974 | hypothetical protein |  |  |  | |
| Autosomal | MVLG_02929 | hypothetical protein |  |  |  | |
| Autosomal | MVLG_03068 | hypothetical protein |  |  |  | |
| Autosomal | MVLG_03601 | hypothetical protein |  |  |  | |
| Autosomal | MVLG_03648 | hypothetical protein |  |  |  | |
| Autosomal | MVLG_03775 | hypothetical protein |  |  |  | |
| Autosomal | MVLG_03809 | hypothetical protein |  |  |  | |
| Autosomal | MVLG_03934 | hypothetical protein |  |  |  | |
| Autosomal | MVLG_04209 | hypothetical protein |  |  |  | |
| Autosomal | MVLG_04374 | hypothetical protein |  |  |  | |
| Autosomal | MVLG_04423 | hypothetical protein |  |  |  | |
| Autosomal | MVLG_04432 | hypothetical protein |  |  |  | |
| Autosomal | MVLG_04452 | hypothetical protein | Pfam | PF05871.5 | ESCRT-II | |
| Autosomal | MVLG_04455 | hypothetical protein |  |  |  | |
| Autosomal | MVLG_04466 | hypothetical protein | Pfam | PF00009.20 | GTP_EFTU | |
| Autosomal | MVLG_04467 | hypothetical protein | Pfam | PF03366.9 | YEATS | |
| Autosomal | MVLG_04468 | hypothetical protein |  |  |  | |
| Autosomal | MVLG_04472 | hypothetical protein | Pfam | PF09793.2 | AD | |
| Autosomal | MVLG_04475 | hypothetical protein |  |  |  | |
| Autosomal | MVLG_04483 | spermidine synthase | Pfam | PF01564.10 | Spermine_synth | |
| Autosomal | MVLG_04484 | hypothetical protein |  |  |  | |
| Autosomal | MVLG_04485 | hypothetical protein | Pfam | PF03124.7 | EXS | |
| Autosomal | MVLG_04487 | hypothetical protein |  |  |  | |
| Autosomal | MVLG_04489 | hypothetical protein |  |  |  | |
| Autosomal | MVLG_04491 | hypothetical protein | Pfam | PF08659.3 | KR | |
| Autosomal | MVLG_04493 | hypothetical protein | Pfam | PF00023.23 | Ank | |
| Autosomal | MVLG_04494 | hypothetical protein |  |  |  | |
| Autosomal | MVLG_04522 | hypothetical protein |  |  |  | |
| Autosomal | MVLG_04551 | hypothetical protein |  |  |  | |
| Autosomal | MVLG_05458 | hypothetical protein |  |  |  | |
| Autosomal | MVLG_05569 | hypothetical protein |  |  |  | |
| Autosomal | MVLG_05572 | hypothetical protein |  |  |  | |
| Autosomal | MVLG_05684 | hypothetical protein |  |  |  | |
| Autosomal | MVLG_05712 | hypothetical protein |  |  |  | |
| Autosomal | MVLG_06013 | hypothetical protein |  |  |  | |
| Autosomal | MVLG_06125 | hypothetical protein |  |  |  | |
| Autosomal | MVLG_06199 | hypothetical protein |  |  |  | |
| Autosomal | MVLG_06212 | hypothetical protein |  |  |  | |
| Autosomal | MVLG_06317 | hypothetical protein |  |  |  | |
| Autosomal | MVLG_06383 | hypothetical protein |  |  |  | |
| Autosomal | MVLG_06499 | hypothetical protein |  |  |  | |
| Autosomal | MVLG_06546 | hypothetical protein |  |  |  | |
| Autosomal | MVLG_06693 | hypothetical protein |  |  |  | |
| Autosomal | MVLG_06713 | hypothetical protein |  |  |  | |
| Autosomal | MVLG_06752 | hypothetical protein |  |  |  | |
| Autosomal | MVLG_06754 | hypothetical protein |  |  |  | |
| Autosomal | MVLG_06941 | hypothetical protein | Pfam | PF07690.9 | MFS_1 | |
| Autosomal | MVLG_06951 | hypothetical protein |  |  |  | |
| Autosomal | MVLG_07023 | hypothetical protein |  |  |  | |
| Autosomal | MVLG_07095 | hypothetical protein |  |  |  | |
| Autosomal | MVLG_07096 | hypothetical protein |  |  |  | |
| Autosomal | MVLG_07171 | hypothetical protein |  |  |  | |
| Autosomal | MVLG_07177 | hypothetical protein |  |  |  | |
| Autosomal | MVLG_07178 | hypothetical protein |  |  |  | |
| Autosomal | MVLG_07222 | hypothetical protein |  |  |  | |
| Autosomal | MVLG_07229 | hypothetical protein | Pfam | PF03583.7 | LIP | |
| Autosomal | MVLG_07231 | hypothetical protein |  |  |  | |
| Autosomal | MVLG_07233 | hypothetical protein |  |  |  | |
| Autosomal | MVLG_07240 | hypothetical protein |  |  |  | |
| Autosomal | MVLG_07255 | hypothetical protein |  |  |  | |
| Autosomal | MVLG_07303 | hypothetical protein |  |  |  | |
| Autosomal | MVLG_07306 | hypothetical protein |  |  |  | |
| Autosomal | MVLG_07313 | hypothetical protein |  |  |  | |
| Autosomal | MVLG_07317 | hypothetical protein |  |  |  | |
| Autosomal | MVLG_07320 | hypothetical protein |  |  |  | |
| Autosomal | MVLG_07324 | hypothetical protein |  |  |  | |
| Autosomal | MVLG_07327 | hypothetical protein |  |  |  | |
| Autosomal | MVLG_07329 | hypothetical protein |  |  |  | |
| Autosomal | MVLG_07333 | hypothetical protein |  |  |  | |
| Autosomal | MVLG_07335 | hypothetical protein |  |  |  | |
| Autosomal | MVLG_07341 | hypothetical protein |  |  |  | |
| Autosomal | MVLG_07342 | hypothetical protein |  |  |  | |
| Autosomal | MVLG_07361 | hypothetical protein |  |  |  | |
| Autosomal | MVLG_07362 | hypothetical protein |  |  |  | |
|  |  |  |  |  |  | |
