# Supplementary material for: Meiotic recombination in the offspring of Microbotryum hybrids and its impact on pathogenicity
Source: BMC Evol Biol. 2020 Sep 17;20:123. doi: 10.1186/s12862-020-01689-2 (PMC7499883; doi:10.1186/s12862-020-01689-2)
Supplement: Supplementary file 3 — Additional file 3. Text file (.docx) listing the used inoculum. Haploid F1-strains used for inoculum. [file 12862_2020_1689_MOESM3_ESM.docx]

**Table S1**. Haploid F1-strains used for inoculum.

| Genotype | Generation | Number of isolates pooled for inoculum | Parental Backcross Strain | Concentration adjusted to |
| --- | --- | --- | --- | --- |
| A1-MSL^hyb^ | F1-hybrid | 31 | A2-MSA^par^ | 4.5*10^7 cells/ml |
| A2-MSA^hyb^ | F1-hybrid | 64 | A1-MSA^par^ | 2.7*10^7 cells/ml |
| A1-MSA^hyb^ | F1-hybrid | 46 | A2-MSA^par^ | 2.25*10^7 cells/ml |
| A2-MSL^hyb^ | F1-hybrid | 50 | A1-MSA^par^ | 2*10^5 cells/ml |
