# Supplementary figures and images for: Meiotic recombination in the offspring of Microbotryum hybrids and its impact on pathogenicity
Source: BMC Evol Biol. 2020 Sep 17;20:123. doi: 10.1186/s12862-020-01689-2 (PMC7499883; doi:10.1186/s12862-020-01689-2)

| A1-MSL | A2-MSL | A1-MSA | A2-MSA | A1-MSL <sup>hyb</sup> | A2-MSA <sup>hyb</sup> | A1-MSA <sup>hyb</sup> | A2-MSL <sup>hyb</sup> |
|--------|--------|--------|--------|-----------------------|-----------------------|-----------------------|-----------------------|
|--------|--------|--------|--------|-----------------------|-----------------------|-----------------------|-----------------------|

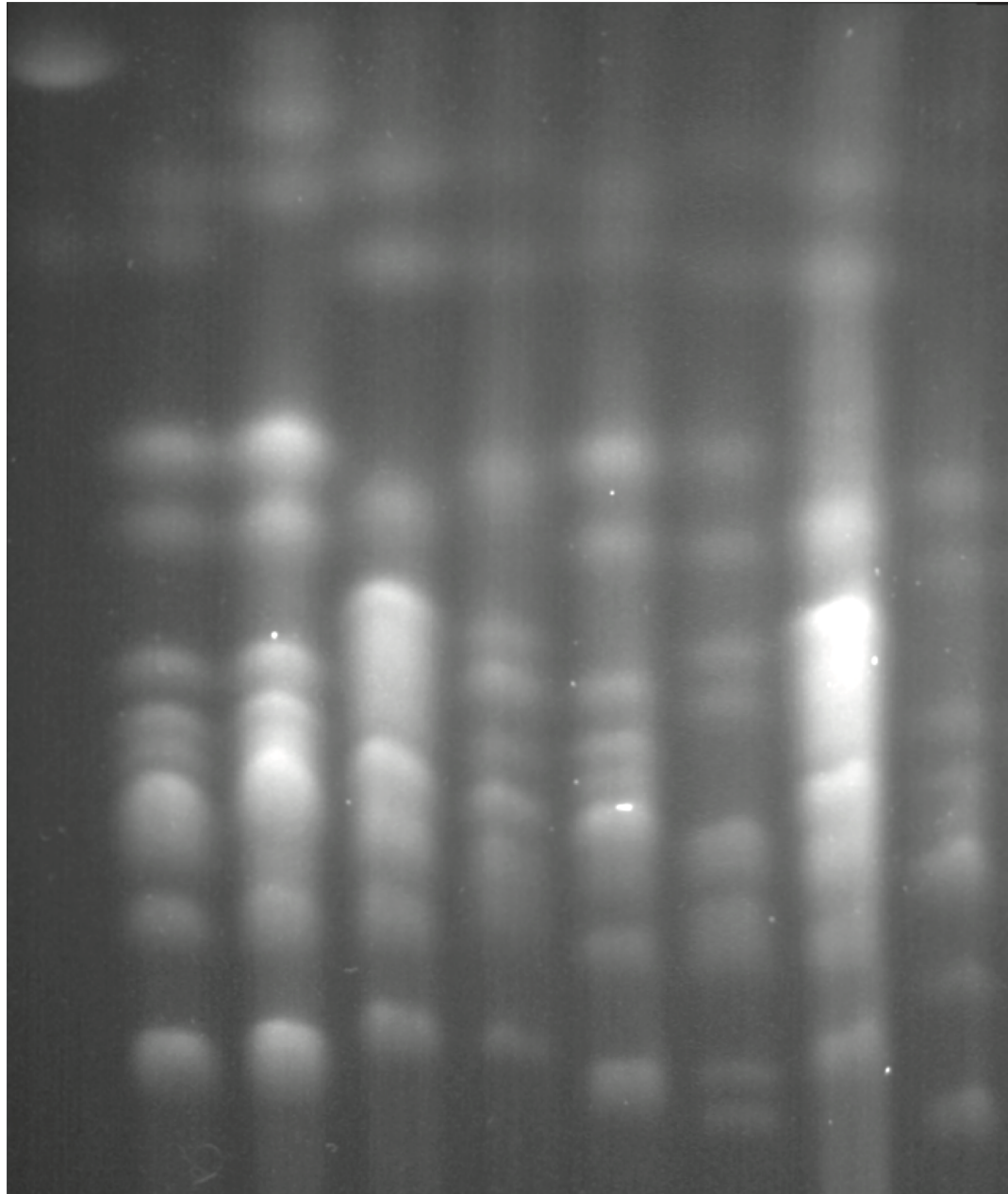

Supplement: Supplementary file 4 — Additional file 4. Gel picture (.pdf) showing the karyotypes of four F1-hybrids and parental strains. Electrophoretic karyotypes for Microbotryum silenes-dioicae and M. silenes-acaulis and F1-hybrids resulting from crosses between the two species. [file 12862_2020_1689_MOESM4_ESM.pdf]
