# Supplementary material for: Meiotic recombination in the offspring of Microbotryum hybrids and its impact on pathogenicity
Source: BMC Evol Biol. 2020 Sep 17;20:123. doi: 10.1186/s12862-020-01689-2 (PMC7499883; doi:10.1186/s12862-020-01689-2)
Supplement: Supplementary file 5 — Additional file 5. Text file (.docx) containing the script for the “global approach”. Detailed script for tracking the origin of reads in hybrids. [file 12862_2020_1689_MOESM5_ESM.docx]

**Text S1 –** *Detailed script for tracking the origin of reads in hybrids*

*Global (gene-independent) k-mer-based approach*

In this approach, we aimed to explain the origin of each read in the hybrid and backcross genomes by its *k*-mer content, using *k* = 15. Taking the equivalence of a DNA sequence and its reverse complement into account, there were approximately 4*^k^*/2 possible distinct *k*-mers. Each *k*-mer was classified into one of four classification types: A *k*-mer was defined to be

- of type 0 if it occurs in neither parent genome,
- of type 1 if it occurs in parent genome MSL and not in parent genome MSA,
- of type 2 if occurs in parent genome MSA and not in parent genome MSL, and
- of type 3 if it occurs in both parent genomes.

To compute the classification type of each *k*-mer, it was not necessary to (partially) assemble the parent genomes. Instead analyses directly proceeded from quality-filtered reads as follows:

Let x be any *k*-mer and *C(x)* the occurrence count of *x* in the set of reads from a given genome. If *c* is the coverage with which the genome was sequenced (e.g., *c* = 20 for 20 x coverage), we expect that *C*(*x*) ≈ c for those *k*-mers *x* that occur once in the genome, *C*(*x*) ≈ 2*c* for those *k*-mers *x* that occur twice in the genome, etc. If *C*(*x*) ≈ 0, we may assume that *x* does not occur in the genome, but is included in some read(s) because of technical errors. From a coverage histogram, we may thus define a threshold *C** and state for the above type classification that “*x* *occurs* in a genome” if *C*(*x*) ≥ *C**. Supplementary Figure S1 shows such a coverage histogram, from which we can infer the sequencing coverage *c* and the optimal threshold *C** (the first minimum to the right of zero).

Once the type (0–3) of each *k*-mer has been determined, we translated each read of the hybrid and backcross genomes into a sequence of types of overlapping *k*-mers. For example, if *k*=3 and the read is AGTCGG, and if the classification types of AGT, GTC, TCG, and CGG are 3, 1, 0, and 1, respectively, then the read is translated into the classification type sequence 3101.

We assigned a likely origin to each read depending on its classification type sequence as follows. We define *N_t_* as the number of occurrences of *t* in the type sequence (*t* = 0,1,2,3), and *N* as the length of the type sequence.

- If there are many zeros in the type sequence, more precisely, if *N*_0_/*N* ≥ *f*_0_, where *f*_0_ is a given threshold fraction (we use 0.8 by default), the read cannot be explained by either genome and is called “of unknown origin” (read type 0).
- Otherwise, if there are fewer zeros in the type sequence, the assigned origin depends on the contained number of 1s, 2s and 3s:
  - If there is a substantial number of 1s, but not a substantial number of 2s in the type sequence (meaning *N*_1_ ≥ *T* but *N*_2_ < *T*, where *T* is another user-defined threshold taking the value of *k*+1 by default), the read is said to originate “from genome MSL” (read type 1).
  - Otherwise, if *N*_1_ < *T* but *N*_2_ ≥ *T*, the read originates “from genome MSA” (read type 2).
  - Otherwise, if *N*_1_ < *T* and *N*_2_ < *T* but *N*_3_ ≥ *T*, the read may be explained “from both genomes” (read type 3).
  - Otherwise, if both *N*_1_ ≥ *T* and *N*_2_ ≥ *T*, the read is called a “hybrid” (read type 4).
  - In the remaining case (all of *N*_1_, *N*_2_, *N*_3_ are < *T*), the read is “of unknown origin” (read type 0).

Extending the original inference from single reads to paired-end reads, we considered the multiset of the two read types and assigned a pair accordingly to a paired-origin class:

- “of unknown origin” (pair type 0) results from read types {0,0},
- “from genome MSL” (pair type 1) results from {1,0}, {1,1}, {1,3},
- “from genome MSA” (pair type 2) results from {2,0}, {2,2}, {2,3},
- “from both genomes” (pair type 3) results from {3,0}, {3,3}, and
- “hybrid” (pair type 4) results from {1,2}, {4,0}, {4,1}, {4,2}, {4,3}, {4,4}.

In the way described, we classified each read and read pair without any need of assembling parent genomes or annotating genes. The classification scheme depends on three parameters (*k*=8, *f*_0_=0.8 and *T*=*k*+1=16). While our results are relatively robust against variations of *f*_0_ and *T*, the choice of *k* is important and was determined by the following considerations. To distinguish both paretnal genomes the most important criterion is that each *k*-mer should have a high probability of being unique in a random sequence of the same length as both parent genomes together. If *L*_1_ and *L*_2_ are the (estimated) lengths of the parent genomes, they contain (approximately) *L* := *L*_1_ + *L*_2_ *k*-mers. In an entirely random sequence, each of the 4*^k^*/2 *k*-mers would thus occur *A* := *L* / (4*^k^*/2) times on average.

Demanding A < 1 is equivalent to *k* > ln(2*L*) / ln(4). With L = 48 MB, this suggests using any *k* ≥ 12. Using slightly larger values than this lower bound may increase resolution between the two parent genomes, but using considerably larger values will result in a larger memory footprint and thus lower performance of the type classification, as well as more type-0 *k*-mers in the reads resulting from sequencing errors. Thus a reasonable range is *k* = 12, …, 16.
