# Supplementary material for: Meiotic recombination in the offspring of Microbotryum hybrids and its impact on pathogenicity
Source: BMC Evol Biol. 2020 Sep 17;20:123. doi: 10.1186/s12862-020-01689-2 (PMC7499883; doi:10.1186/s12862-020-01689-2)
Supplement: Supplementary file 6 — Additional file 6. Diagram (.pdf) representing coverage histogram for the “global approach”. Example for a coverage histogram from which sequencing coverage c and the optimal threshold C* was inferred. [file 12862_2020_1689_MOESM6_ESM.docx]

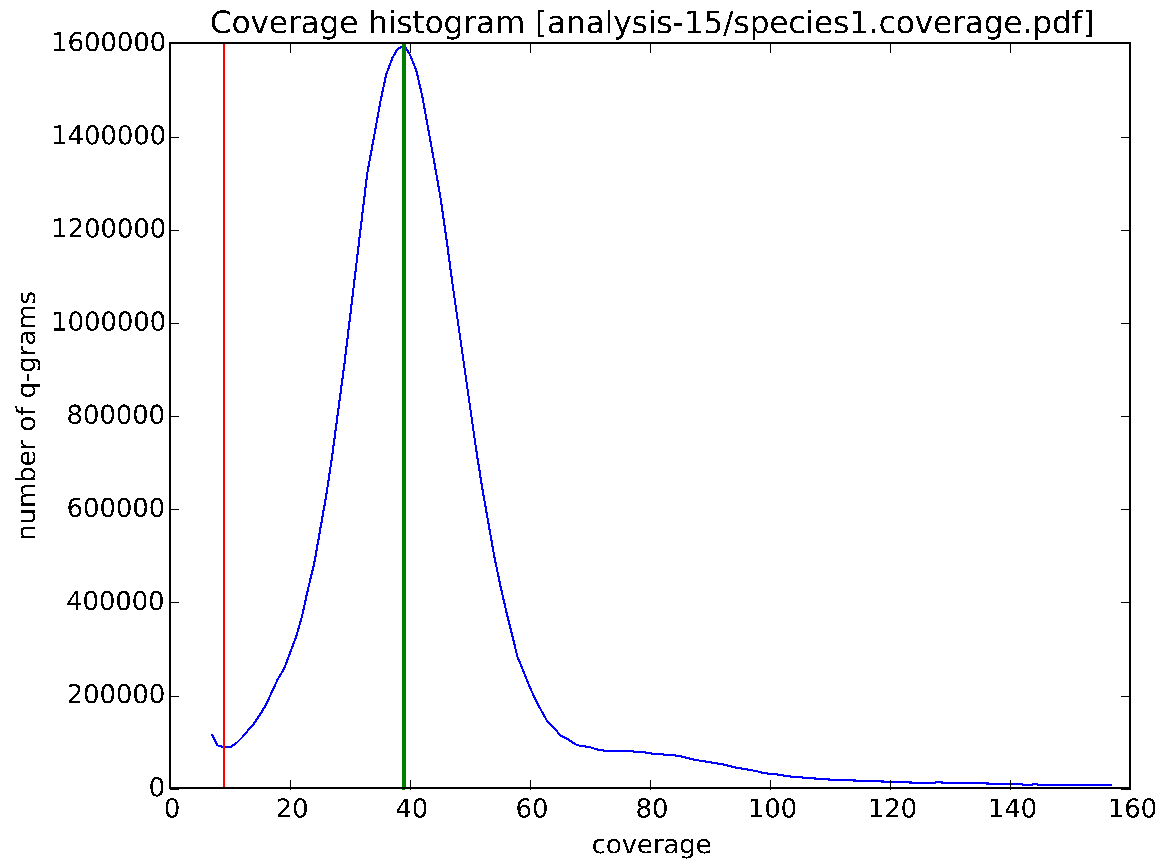


**Figure S1**: Coverage histogram, from which sequencing coverage *c* and the optimal threshold *C** was inferred.
